# Enhancing Respiratory Disease Surveillance to Detect COVID-19 in Shelters for Displaced Persons, Thailand–Myanmar Border, 2020–2021

**DOI:** 10.3201/eid2813.220324

**Published:** 2022-12

**Authors:** Barbara Knust, Nuttapong Wongjindanon, Aye Aye Moe, Lasantha Herath, Wiphan Kaloy, Thin Thin Soe, Preeyalak Sataranon, Htay Min Oo, Kyaw Zaw Myat, Zarni Win, Myo Htet, Min Htike, Banjong Sudhiprapha, Aye Aye Pyone, Thet Phyo Win, Hnin Zaw Win, Pongpun Sawatwong, Wanitda Watthanaworawit, Clare Ling, Sajith Gunaratne, Sai Aung Lynn, Leena Bhandari, Francois Nosten, Beth Skaggs

**Affiliations:** Centers for Disease Control and Prevention, Nonthaburi, Thailand (B. Knust, N. Wongjindanon, P. Sawatwong, B. Skaggs);; International Rescue Committee, Mae Sot, Thailand (A.A. Moe, T.T. Soe, P. Sataranon, K.Z. Myat, Z. Win, M. Htet, M. Htike, B. Sudhiprapha, H.Z. Win);; Malteser International, Mae Sariang, Thailand (L. Herath, W. Kaloy, A.A. Pyone, T.P. Win);; Committee for Coordination of Services to Displaced Persons in Thailand, Mae Sariang (H.M. Oo);; Shoklo Malaria Research Unit, Mahidol University, Mae Sot (W. Watthanaworawit, C. Ling, F. Nosten);; University of Oxford Centre for Tropical Medicine and Global Health, Oxford, UK (C. Ling, F. Nosten);; International Organization for Migration, Bangkok, Thailand (S. Gunaratne, S.A. Lynn, L. Bhandari)

**Keywords:** COVID-19, infectious disease surveillance, refugees, coronavirus disease, SARS-CoV-2, severe acute respiratory syndrome coronavirus 2, viruses, respiratory infections, zoonoses, displaced persons, Thailand, Myanmar

## Abstract

We developed surveillance guidance for COVID-19 in 9 temporary camps for displaced persons along the Thailand–Myanmar border. Arrangements were made for testing of persons presenting with acute respiratory infection, influenza-like illness, or who met the Thailand national COVID-19 Person Under Investigation case definition. In addition, testing was performed for persons who had traveled outside of the camps in outbreak-affected areas or who departed Thailand as resettling refugees. During the first 18 months of surveillance, May 2020–October 2021, a total of 6,190 specimens were tested, and 15 outbreaks (i.e., >1 confirmed COVID-19 cases) were detected in 7 camps. Of those, 5 outbreaks were limited to a single case. Outbreaks during the Delta variant surge were particularly challenging to control. Adapting and implementing COVID-19 surveillance measures in the camp setting were successful in detecting COVID-19 outbreaks and preventing widespread disease during the initial phase of the pandemic in Thailand.

SARS-CoV-2, the causative agent of COVID-19, is a highly transmissible coronavirus that easily infects persons living in high-density environments, especially when distancing is difficult and fresh air ventilation is limited. Numerous COVID-19 outbreaks in such settings have been described (e.g., nursing homes, prisons, cruise ships); attack rates have reached and often exceeded 20% (*1*–*4*). Crowded and resource-limited conditions make refugee and displaced persons’ shelters, or camps, particularly prone to communicable disease outbreaks, and numerous previous examples of residents being affected by waterborne (*5*,*6*), vectorborne (*7*,*8*), and respiratory pathogens (*9*,*10*) have been documented. From the start of the COVID-19 pandemic, many experts have raised concerns about the particular risk in the setting of temporary camps for displaced persons (*11*,*12*), and outbreaks have been reported among displaced populations in several countries, including Bangladesh (*11*), Greece (*13*), and Brazil (*14*).

Early detection is key to rapid and successful response efforts in such environments, and existing syndromic surveillance systems can be successfully adjusted to include COVID-19 screening. In this study, we describe the development of an enhanced surveillance program to detect and respond to COVID-19 in displaced persons’ camps on the Thailand–Myanmar border.

Currently, 9 distinct camps in 4 Thailand provinces along the Myanmar border exist (Mae Hong Son, Tak, Kanchanaburi, and Ratchaburi), with a total population of ≈92,000 (*15*). Nongovernmental organizations (NGOs) provide healthcare following guidance of international standards (*16*). Patients whose conditions cannot be managed in the camp setting are referred to Thai Ministry of Public Health (MOPH) facilities for specialized care as needed. The Committee for Coordination of Services to Displaced Persons in Thailand (CCSDPT) consists of 13 NGOs that work to implement and maintain programs and services for refugees (*17*), including health programs. A Health Information System (HIS) for general disease surveillance and reporting was introduced in 2001 and is active across all 9 camps, overseen by CCSDPT. Weekly reports are submitted to the United Nations High Commissioner for Refugees Integrated Refugee Health Information System and shared with Thai MOPH (*18*,*19*). Notifiable disease conditions include severe respiratory disease caused by influenza or coronaviruses and with classifications for immediate notification to the system.

After COVID-19 was declared a pandemic by the World Health Organization (WHO) in mid-March 2020 (*20*), CCSDPT and the United Nations High Commissioner for Refugees developed a coordinating mechanism for COVID-19 preparations and response in the camps (*21*), which included a Surveillance and Outbreak Response Pillar group that developed an enhanced surveillance system. In this study, we describe this system’s development and its progress in the first 16 months after inception (May 2020–October 2021). Existing surveillance to detect acute respiratory infection (ARI) and influenza-like illness (ILI) was used as a platform for COVID-19 testing, which might have enhanced SARS-CoV-2 detection within this population. We also briefly describe the COVID-19 outbreaks (defined as >1 laboratory-confirmed case) detected through this system.

## Materials and Methods

### Surveillance Guidelines and Procedures

The Surveillance Pillar working group reviewed existing Thai MOPH guidance (*22*) and built consensus plans for essential control and response areas. Plans were written into surveillance guidelines and shared with local and national public health entities for review and approval (Appendix). The Thai MOPH and Thai Ministry of Interior (MOI) reviewed the guidelines and procedures described. The camp surveillance guidelines have the following sections, each of which we describe briefly.

#### Prevention of COVID-19 Introduction through Movement Controls and Social Mobilization

Unauthorized entry into the camps was not permitted according to MOI requirements. All persons entering camps were screened for signs of COVID-19, such as elevated temperature or obvious signs of illness, and asked about symptoms. Risk communication and community engagement campaigns were enacted in the camps to promote awareness of COVID-19 and encourage sanitation and disease prevention measures such as handwashing, social distancing, and mask use.

#### Surveillance Case Definitions and Case Reporting

All patients receiving inpatient or outpatient services at camp health clinics were screened for respiratory symptoms and history of travel outside the camp. We set criteria for reporting suspected or confirmed cases according to MOPH (*22*) and HIS general infectious disease case definitions (*18*). Patients were tested if they met the national case definition for a Person Under Investigation (PUI) (*21*). In addition, patients who met the existing HIS case definitions for ILI and ARI (Appendix) were tested for COVID-19. Testing for patients meeting the ILI or ARI case definitions was conducted on a voluntary basis. Initially, 100% of patients with ILI and 10% of patients with ARI were offered testing, but as COVID-19 incidence increased in Thailand and testing capacity expanded, larger proportions of these patients were offered testing.

Camp residents were resettling in other countries as refugees throughout the surveillance period. As part of the requirements for international travel, all resettling refugees were tested using reverse transcription PCR (RT-PCR) shortly before their departure.

In accordance with MOPH requirements, District Health Officers were immediately notified of all persons meeting the PUI case definition. All laboratory-confirmed COVID-19 cases were reported through the official MOPH COVID-19 system and in parallel through the existing HIS surveillance system (Appendix). At the start of surveillance in the camps, COVID-19 cases had not yet been detected. Because a single laboratory-confirmed COVID-19 case necessitated outbreak response measures, an outbreak of COVID-19 was defined as any new detection of a case that was not associated in time or place with other COVID-19 cases in the same camp. An outbreak was considered finished after 28 days (2 incubations periods of 14 days) had passed with no new confirmed cases.

#### Care Provision

PUIs were isolated at a designated facility at the camp or were referred to designated district hospitals while COVID-19 testing was pending, depending on the availability of referral hospital beds, symptom severity, and local situations. Patients meeting ARI or ILI case definitions were advised on social distancing measures and asked to self-isolate at their house while tests were pending. Confirmed COVID-19 case-patients were isolated either in camp isolation units or referred to district hospitals according to MOPH standards (*23*). As the number of confirmed cases increased in an outbreak, healthcare providers developed additional community isolation units for asymptomatic and mildly symptomatic patients; when the case count exceeded the capacity of these community isolation units, house isolation for asymptomatic and mild cases was initiated.

#### Laboratory Testing

Healthcare staff collected nasopharyngeal swabs according to national protocols (*22*); swabs were placed in commercial transport media and transported to the laboratory following recommended cold chain requirements. As per national reporting requirements, positive results were immediately reported to the MOPH district health office and to the NGO providing healthcare.

Starting in July 2021, camp staff used commercial antigen test kits (ATKs) authorized by the Thai Food and Drug Administration from 3 manufacturers (Abbott, https://www.abbott.com; Roche, https://www.roche.com; Humasis, http://www.humasis.com). ATK sensitivity, as reported through real-world testing, varied from 56% to 65%, and specificity varied from 79% to 100% (*24*). ATK-positive results were recorded as probable cases, but only RT-PCR–positive cases were recorded as confirmed and reported to MOPH. Camp medics performed RT-PCR testing after antigen testing if a patient had a negative ATK result but had symptoms consistent with COVID-19 or if the patient was a close contact of a confirmed SARS-CoV-2–positive person (Appendix). Camp staff collected specimens and performed the antigen test in camp laboratory settings.

#### Case Investigation

When a PUI was identified, camp-based investigation teams interviewed the patient to complete the national Case Investigation Form as per MOPH requirements (*22*). To the extent possible, the teams documented the PUI’s exposures before and after disease onset.

#### Contact Tracing

Camp-based contact tracing teams began contact tracing as soon as a PUI was identified, because laboratory confirmation required 3–5 days in some remote camps. High-risk and low-risk contacts were defined according to Thai MOPH guidelines (*22*).

#### Quarantine

Quarantine was used for 2 groups in the camp setting: close contacts of confirmed cases and persons with a history of travel outside the camp in the past 14 days (travel quarantine). Quarantine was administered at a designated facility or in the person’s house, depending on availability of resources. For both types, persons were notified of their quarantine status and received instructions on social distancing measures. Support was provided in the form of meals, medications, daily living supplies, and other necessary services. Persons were checked by camp-based staff daily, and RT-PCR testing of a nasopharyngeal swab specimen was performed 1–2 times during the 14-day follow-up period.

#### Active Case Finding

During outbreak investigations, persons in the general community who were not known close contacts of cases were offered testing as a means to identify additional cases and chains of transmission within the community. Depending on resources, RT-PCR or ATK testing was used.

##### Laboratory Methods

Given the geographic distribution of the 9 camps (*15*), SARS-CoV-2 RT-PCR testing was performed in 5 different Thai MOPH-approved laboratories: Shoklo Malaria Research Unit, Tak Province; CDC-Thailand Division of Global Health Protection Laboratory, Nonthaburi Province; Paholpolpayuhasena Laboratory, Kanchanaburi Province; Sri Sam Wan Provincial Laboratory, Mae Hong Son Province; and IOM Migration Health Division, Tak Province. As per Thai MOPH requirements, all laboratories authorized to perform SARS-CoV-2 RT-PCR participated in a national quality assurance program and used primers, probes, and reagents that are authorized through WHO Emergency Use Listing procedures.

##### Data Collection and Analysis

Health NGOs at each camp compiled weekly surveillance metrics reports, which described numbers of persons tested and numbers in quarantine. When an outbreak was detected, additional information was shared summarizing the outbreak dynamics and case report information. Weekly summaries were combined into a database and analyzed to provide descriptive statistics using the Power Bi statistical analysis software (Microsoft, https://www.microsoft.com). We included data reported during May 1, 2020–October 29, 2021 in the analysis.

##### Community Engagement and Training

Health NGOs recruited camp residents and trained them as community response staff in the COVID-19 control and prevention response. Refresher trainings were held regularly to share new updates on MOPH recommendations, requirements, and procedures. Simulation exercises were conducted to practice various scenarios involving the healthcare team and the wider community.

##### Funding Sources, Nonresearch Determination Status

Funding for the surveillance and outbreak response activities was provided by the US Centers for Disease Control and Prevention COVID-19 response funds, with additional support provided by the US Department of State Bureau for Population, Refugees, and Migration; the European Union; Malteser International; and International Rescue Committee. The Shoklo Malaria Research Unit is part of the Wellcome Trust Mahidol University Oxford Tropical Medicine Research Unit, which is funded by the Wellcome Trust 220211. For the purpose of open access, the author has applied a CC BY public copyright license to any author accepted manuscript version arising from this submission. Surveillance activities were determined to be public health response and not research by the Centers for Disease Control and Prevention, International Rescue Committee, and Malteser International COVID-19 response oversight committees.

## Results

**Figure 1:**
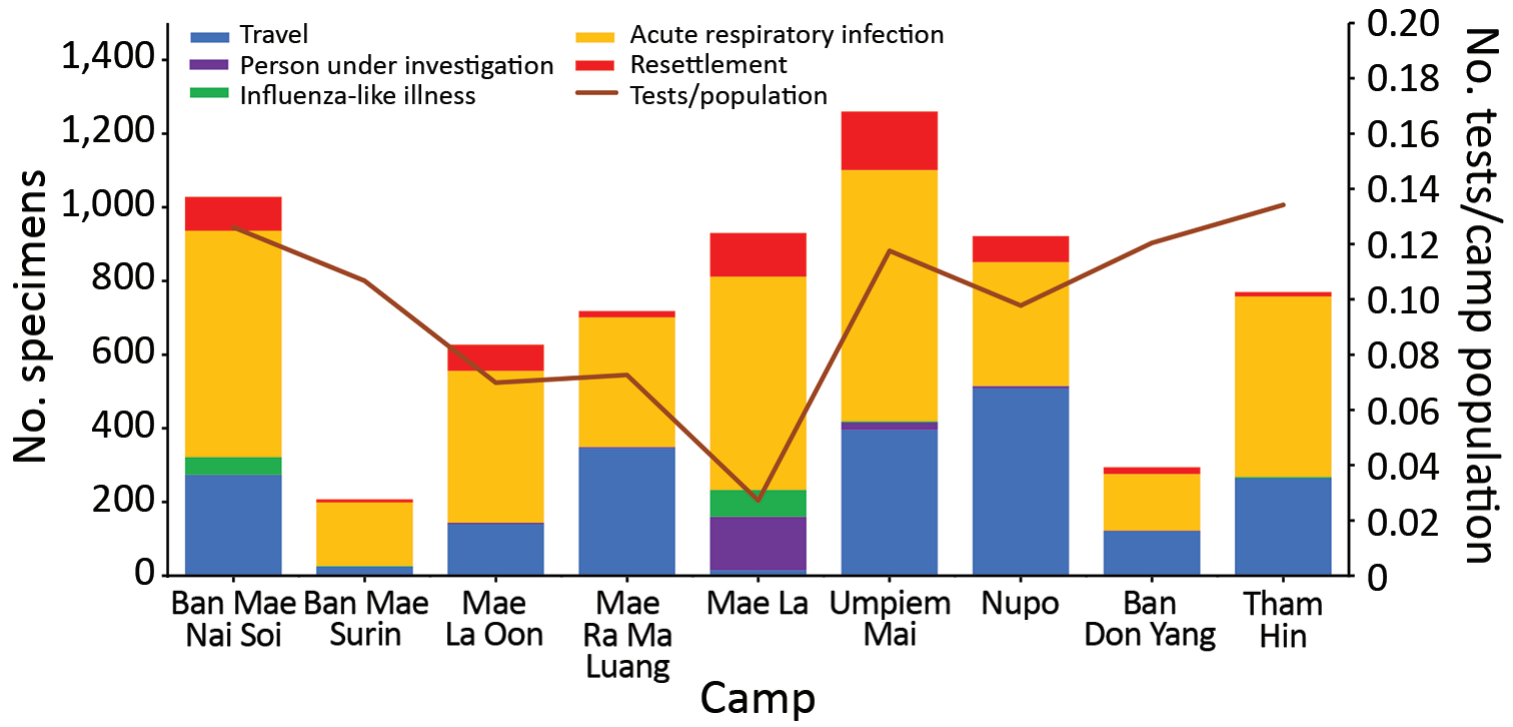
Total number of nasopharyngeal swab specimens tested for SARS-CoV-2 by reverse transcription PCR by camp and reason for testing as part of enhanced surveillance for COVID-19 in displaced persons’ shelters, Thailand–Myanmar border, May 2020–October 2021. Travel indicates persons who had traveled outside of the camp in the previous 14 days. Resettlement refers to persons tested before international travel to a third country as part of refugee resettlement. For reference, population sizes of each camp are given in Table 1.

During May 2020–October 2021, camps submitted a total of 6,190 specimens collected as part of enhanced surveillance (i.e., not as part of an outbreak investigation) (Figure 1). Of these, 2,091 (34%) were specimens submitted from persons in travel quarantine, 3,791 (61%) were patients with ARI, 129 (2%) were patients with ILI, and 179 (3%) were PUIs. In addition, 13,586 specimens were collected as part of outbreak response activities; 4,350 (32%) were specimens from close contacts and 9,236 (68%) were specimens collected in the community as part of active case finding. Surveillance tests performed per person varied from 0.02 in Mae La to 0.13 in Tham Hin.

**Table 1:** Summary of COVID-19 surveillance and outbreaks detected at 9 displaced persons’ shelters, Thailand–Myanmar border, May 2020–October 2021*

Indicator	Prelockdown, mean (95% CI)	Postlockdown, mean (95% CI)	SARIMA parameters
Total sexual violence cases	2,387 (2,289–2,485)	5,269 (4,289–6,250)	(4,1,0) x (1,1,0,12)
Rape	1,037 (989–1,085)	1,801 (1,576–2,028)	(0,1,0) x (1,0,0,12)
Rape-PEP	628 (603–653)	910 (814–1,007)	(1,1,1)
Rape-STI treatment	745 (714–776)	1,115 (980–1,249)	(0,1,0)

*Trends were measured during January 2015–June 2020. PEP, postexposure prophylaxis for HIV; SARIMA, seasonal autoregressive integrated moving average; STI, sexually transmitted infection.

A total of 14 COVID-19 outbreaks were detected in the camps during the 18-month surveillance period for a total of 1,342 cases reported (Table 2). In 10 outbreaks, <10 cases were identified; 5 outbreaks were limited to a single case. Five outbreaks were detected by testing done during travel quarantine, and 9 were detected by testing patients with ARI symptoms. The index cases for all 14 outbreaks were identified and laboratory confirmed. Probable introduction of COVID-19 into the camp was estimated to have occurred 1–2 weeks before detection for all outbreaks.

**Table 2:** COVID-19 outbreaks in 9 displaced persons’ shelters, Thailand–Myanmar border, with cumulative number of cases as of October 31, 2021*

Camp	Population†	Surveillance start date (wk)	No. surveillance specimens tested‡	PUI	Persons with ARI	Persons with ILI	Persons under travel quarantine	No. outbreak response specimens tested§	No. outbreaks detected
Ban Mae Nai Soi	8,152	2020 Aug 1 (wk 31)	936	0	614	48	274	NA	0
Ban Mae Surin	1,939	2020 Aug 1 (wk 31)	199	0	172	2	25	NA	0
Mae La Oon	8,971	2020 May 9 (wk 19)	556	4	412	0	140	379	1
Mae Ra Ma Luang	9,884	2020 May 9 (wk 19)	701	3	352	0	346	195	1
Mae La	34,211	2020 Aug 1 (wk 31)	812	145	579	73	15	7,151	2
Umpiem Mai	10,715	2020 Aug 1 (wk 31)	1,101	20	682	3	396	3,236	5
Nupo	9,429	2020 Aug 1 (wk 31)	851	6	336	0	509	177	2
Ban Don Yang	2,440	2021 Mar 8 (wk 10)	276	1	154	0	121	127	2
Tham Hin	5,738	2020 Aug 29 (wk 35)	758	0	490	3	265	2,136	2
Total	91,479	NA	6,190	179	3,791	129	2,091	13,401	15

*ARI, acute respiratory illness; ILI, influenza-like illness; NA, not applicable; PUI, persons under investigation; wk, epidemiological week. †Population verified by United Nations High Commissioner for Refugees and The Border Consortium as of November 2020. ‡Surveillance specimens were collected from persons meeting the case definition criteria for PUI, ARI, or ILI, and from persons who had returned from travel outside the camp in the previous 14 days and were under quarantine. §Outbreak response specimens include specimens collected from close contacts of confirmed cases and active case finding in the community. Totals may not include some specimens that were tested by the Thai Ministry of Public Health during first outbreaks in Umpiem Mae and Tham Hin camps.

The first outbreak with >10 cases was at Tham Hin camp, Ratchaburi Province, in April 2021. At the time, Alpha variant was the predominant strain in Thailand. Case investigation found that the index case-patient had been visited by family members who circumvented travel quarantine. The index case-patient was a religious leader and had close contact with nearly 100 persons during the infectious period. The large number of high-risk close contacts overwhelmed quarantine facilities, so a house quarantine approach was started. Community isolation facilities were used for all close contacts who tested positive, regardless of clinical symptoms. A lockdown of the camp was instituted for 4 weeks after detection of this outbreak, in which only 1 designated person in each nonquarantined household was allowed to move about the camp to pick up food rations and other necessary supplies. After 6 weeks of intensive contact tracing, 110 total confirmed cases were identified, and the outbreak was considered controlled.

The number of outbreaks detected increased during August 2021 and continued until the time of this report in November 2021, after the wave of community transmission across Thailand from the Delta variant (Table 2). When outbreaks were detected in camps and confirmed by RT-PCR, active case-finding using ATKs was performed. Movement restrictions in certain camp sections were implemented on the basis of evidence of transmission in the general community. As the outbreaks grew in size, house isolation was implemented for patients with asymptomatic or mild infections, and teams were deployed to provide hygiene materials and daily check-ups on clinical status. Contact tracing, home quarantine, and testing of high-risk contacts continued.

## Discussion

Over 18 months during 2020–2021, a novel COVID-19 surveillance system was launched in 9 refugee camps along the Thailand–Myanmar border; this system tested >6,000 specimens and detected 15 outbreaks. The system incorporated national surveillance recommendations and adapted them for the camp-based setting, where human and physical resources are more limited than in other parts of Thailand. To account for these limitations, laboratory testing was expanded and offered to patients demonstrating symptoms of ARI and ILI to increase sensitivity of the surveillance to detect COVID-19. In total, 9 outbreaks were detected through testing of symptomatic persons at the camps’ clinics. In addition, testing of residents under quarantine after travel outside the camp detected 5 outbreaks during this period. This system operated in parallel with and was complementary to the existing camp HIS and national COVID-19 surveillance systems, and all cases were reported in the relevant systems.

Although direct comparisons of COVID-19 surveillance across different humanitarian settings is challenging because of differences in disease detection, reporting, and local outbreak conditions, reports from other countries offer other examples of functional case detection. In Greece, during the initial 9 months of the pandemic in 2020, a total of 25 outbreaks were detected in 39 refugee and asylum-seeker reception facilities with a total population of ≈60,000 (*13*). In Yemen, a community-based surveillance system generated 91 alerts and detected 5 COVID-19 outbreaks in an internally displaced population of 1,806 persons over a 5-month period (*25*). At Cox’s Bazar in Bangladesh, 3,084 cumulative cases had been reported out of 63,776 total tests performed as of September 2021, for a positivity rate of ≈4.8% (*26*).

The establishment and conduction of laboratory surveillance in the camps themselves was critical. The remote locations of several camps necessitated special transportation arrangements to preserve cold chain requirements and reach laboratories in appropriate times. Relying on testing through official channels would have led to delays in detection and outbreak response because of the challenges in transport and the more stringent PUI case criteria for testing by MOPH laboratories. Some patients who were tested met PUI criteria, but they were a small subset (n = 146), and no outbreaks were detected from PUI testing. Additional patients would possibly have met PUI criteria, but their exposure risk was either not assessed or they were not forthcoming about potential exposure risks.

**Figure 2:**
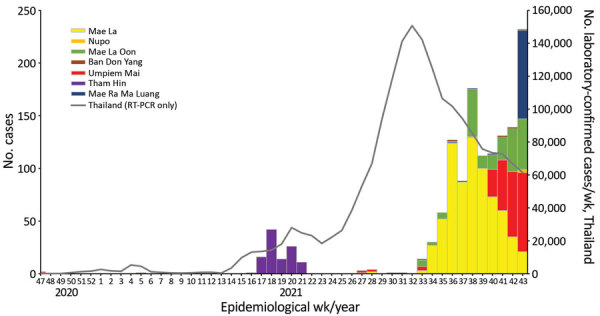
Epidemiologic curve of the total number of laboratory-confirmed COVID-19 cases per week by displaced person camp, Thailand–Myanmar border, November 8, 2020–October 31, 2021. For reference, population sizes of each camp are given in Table 1. RT-PCR, reverse transcription PCR.

Thailand did not have widespread community transmission until mid-2021, when the Delta variant became the predominant strain. This timing afforded camp-based healthcare providers time to plan, recruit and train staff, and bring the enhanced surveillance system into action. During July–October 2021 alone, 11 outbreaks were detected. This number corresponded roughly to the high level of community transmission that was seen across Thailand during that time (Figure 2). In November 2021, several camps were experiencing growing outbreaks. Community resistance to distancing measures, isolation, and testing has been a factor in controlling spread and has been similarly described in other refugee communities (*27*). To build support in this community, risk communication and community engagement activities are ongoing.

A previous modeling paper by Gilman et al. (*28*) identified that the application of control measures, such as efficient isolation of infected persons, use of face masks, and limiting movement of camp residents between sectors, would be effective in limiting COVID-19 transmission. Similar control measures were applied and appeared to have an effect in Tham Hin camp. The outbreak during April–May 2021 started from multiple contacts of an infected person, which nearly overwhelmed the quarantine facilities that had been prepared. Speedy adjustment to the situation and the decision to use house quarantine for close contacts was critical to ensure that existing facilities could accommodate persons who tested positive. Active case finding through systematic screening by camp sections served to identify and stop unknown chains of transmission. Diligent contact tracing, community participation, provision of support to quarantined and isolated persons through food aid, and daily healthcare visits to quarantined households limited transmission; the outbreak was declared over with a total of 110 cases detected after 2 months.

Commercial ATKs were not approved for use in Thailand until July 2021 but were rapidly adopted as an essential tool because of their lower cost, rapid turnaround time, and lack of cold chain requirements. ATKs were particularly helpful because diagnostic laboratories were often distant from the camps, and sample transport and processing required 3–5 days. As an example, a close contact with a positive ATK result could be rapidly isolated and contact tracing could begin while RT-PCR results were pending. False-negative results, however, are commonly experienced with ATK tests because of their lower sensitivity, so RT-PCR testing was still relied upon for confirmation.

The enhanced surveillance system was subject to several limitations. Camp medical staff did not complete comprehensive examination forms for patients seeking care at the ARI clinic, so we could not evaluate whether patients were correctly classified as ARI, ILI, or PUI. Because testing of patients in the ARI clinic was voluntary, uptake varied and the number of tests performed might not accurately reflect the overall incidence of ARI and ILI; some COVID-19 cases could have been missed. Surveillance testing per population was nearly 5-fold greater in Tham Hin camp than in Mae La camp; this difference was related to several factors, including community acceptance of testing.

Similarly, the number of tests performed on persons in travel quarantine might not indicate the total number of persons who returned to a particular camp. Lags in test results and reporting could have caused discrepancies in the total number of COVID-19 cases described in the camps in this study compared with official numbers reported by Thai MOPH. Because ATKs are not as highly sensitive or specific as RT-PCR testing, some COVID-19 cases could have been missed, and the incidence of COVID-19 in the camps might be underestimated.

Despite many humanitarian settings having robust surveillance, more published reports are needed that describe such systems (*29*). A review of the literature covering COVID-19 surveillance found 2 other studies that describe implementation and adaptation to a humanitarian setting, in Yemen and Sudan (*25*,*30*). In Sudan, healthcare providers were trained as rapid response teams (*30*), whereas in Yemen a community-based surveillance system approach was used (*25*). The surveillance system we describe includes elements of community- and healthcare-based surveillance, in which community-based assistants perform contact tracing, identify persons with recent travel history, and refer persons with compatible illness for testing. In addition, our enhanced surveillance system also has an element based in existing clinics, with testing provided for persons experiencing symptoms of ARI and ILI.

COVID-19 surveillance in refugee, migrant, and displaced person populations continues long-term as successive waves of SARS-CoV-2 transmission continue worldwide and vaccine campaigns gradually increase their coverage. Refugees and displaced persons frequently have reduced access to public health services because of language barriers, location in remote areas, and healthcare systems that exclude noncitizens or unofficial residents. Because mobile populations might be more likely to move informally within a country or internationally, establishing surveillance to detect pathogens of international significance and extending national surveillance systems to these groups are vital. The enhanced surveillance developed in displaced persons’ shelters on the Thailand–Myanmar border is one such example and has provided a functional solution to this ongoing challenge.

Appendix Additional information about enhancing respiratory disease surveillance to detect COVID-19 in shelters for displaced persons, Thailand–Myanmar border, 2020–2021.
