# Supplementary material for: Enhancing Respiratory Disease Surveillance to Detect COVID-19 in Shelters for Displaced Persons, Thailand–Myanmar Border, 2020–2021
Source: Emerg Infect Dis. 2022 Dec;28(Suppl 1):S17–25. doi: 10.3201/eid2813.220324 (PMC9745244; doi:10.3201/eid2813.220324)
Supplement: Appendix — Additional information about enhancing respiratory disease surveillance to detect COVID-19 in shelters for displaced persons, Thailand–Myanmar border, 2020–2021. [file 22-0324-Techapp-s1.pdf]

Article DOI: https://doi.org/10.3201/eid2813.220324

Enhancing Respiratory Disease Surveillance to Detect COVID-19 in Shelters for Displaced Persons, Thailand–Myanmar Border, 2020–2021

Appendix

Displaced Persons in Thailand: Guidelines for Prevention, Surveillance, Investigation, and Mitigation of COVID-19

Prepared with input from: Thai Ministry of Public Health—Department of Disease Control, Division of Epidemiology; Thai Ministry of Public Health—Tak Provincial Health Office; Thai Ministry of Public Health—Mae Hong Son Provincial Health Office; Thai Ministry of Public Health—Kanchanaburi Provincial Health Office; Thai Ministry of Public Health—Ratchaburi Provincial Health Office; Thai Ministry of Interior—Operation Center for Displaced Persons; International Rescue Committee; Malteser International; International Organization for Migration; World Health Organization; United States Centers for Disease Control and Prevention, Thailand MOPH – U.S. CDC Collaboration (TUC).

Adapted from internal guidance developed by CCSDPT COVID-19 Surveillance Pillar, August 2021.

1. **Prevention of COVID-19 introduction into the camps**

i. **Entry Screening**—No unauthorized entry or exit is allowed. Camp Commanders and their staff monitor entry and exit of authorized persons to shelters at official gates. MOI staff will register the incoming persons and notify health Non-Governmental Organizations (NGOs) to ensure entry screening will be performed. For some incoming persons with travel history outside of Thailand, travel quarantine will be instated (see section 7, Quarantine). Temperature and symptom screening will be performed with additional support provided by NGOs for necessary supplies, equipment, and training.

Procedures for entry screening are as follows: all personnel who enter will be asked to wash their hands and have their temperature screened with a non-contact thermal device. Visitors who are sick or measure a temperature greater than 37.3 C are not allowed to enter to the camps without further evaluation. If a shelter resident is sick or registers a temperature greater than 37.3 C, they would not be allowed entry. The Camp Commander staff member will ask the relevant agency accompanying the resident's return to perform further evaluation without bringing the person into the camp. Residents returning from care at a district hospital outside the camp would be handled on a case-by-case basis. If a temperature is detected in a person who is returning to camp unaccompanied, MOI should contact district hospital or District Health Office as per district surveillance procedures for PUIs and would not be allowed entry.

If a person with 37.3 C temperature is detected, they will be referred to the Health NGO for secondary screening, and they will evaluate the person to determine if they meet PUI case definition criteria (see section 2).

All Health NGO staff are routinely screened for respiratory symptoms (including fever) before leaving offices before departure to the shelters and are not permitted to go to the shelter if any symptoms are detected. They are screened again at the camp's' gate. NGO staff are required to wear cloth masks or surgical masks while at the shelter, to reduce the potential for asymptomatic transmission to the camp population.

ii. **Social Mobilization**—NGOs and community groups will work to disseminate messages to the general population about the importance of remaining within the camp, information about the signs and symptoms of COVID-19, ways to report illness, and hygiene measures. MOPH or World Health Organization consistent messages will be adapted and translated into local languages.

iii. **General Hygiene and Social Distancing measures**—NGOs will work to set up handwashing stations at all offices onsite and evaluate general procedures within the camp that could lead to congregation of persons (i.e., mass events for social or service distribution) and take measures to limit crowds, in coordination with shelter resident leaders. Events and meetings will be limited during this time, and specific limitations in

terms of number of participants in any form of meeting will be set by the Camp Committees and/or the Camp Commanders which reflect the current risk level.

iv. **Camp Access**— Travel restriction (entry and exit) is implemented for all camp residents regarding ‘state of emergency declaration’ for COVID-19 prevention and control. Camp Commanders will take steps to reduce visitor access to the camps, which includes the following: All visitors, NGOs staff that do not have essential function at the camps, and donors with recent travel history outside of Thailand are not allowed to enter until further notice, with exception of critical surge healthcare staff required to contain an outbreak within one or more of the shelters. For health care workers with history of travel outside of the province, 14-day quarantine may not be required, but screening procedure is still needed (body temperature scan, asked for history of risk factors) as determined by PHO. All NGOs will continue to provide essential services, which includes medical and food delivery, and entities who have been identified as involved in outbreak response (Ministry of Public Health, NGO, etc.).

v. Camp commanders will perform patrols of the grounds to prevent and detect unauthorized movements.

2. Surveillance

i. **MOPH PUI Case definitions**

MOPH PUI Case Definitions and Guidelines for Surveillance and Investigation will be used to identify PUIs in the displaced person shelters. Current surveillance within the shelters is administered through the CCSDPT Health Information System (HIS). Report of any confirmed COVID-19 cases and PUIs must be made by following existing CCSDPT system (Weekly Outbreak Alert Form, OAF) where cases of notifiable diseases are reported promptly.

a. Scenario 2.1: Suspected patients with any of the following signs and symptoms: history of fever or body temperature ≥37.5 C, cough, runny nose, sore throat, loss of the sense of smell (anosmia), loss of the sense of taste (ageusia), rapid breathing (tachypnea), shortness of breath, difficulty breathing, diarrhea, eye redness, and/or rash;

1) Having any of the following exposure risks in the past 14 days: 1.1) Travel history to/ from or reside in areas with ongoing local transmission of COVID-19 in the past month, 1.2) Exposure to suspected or confirmed cases of COVID-19, 1.3) Has visited crowded places or large gatherings gathering where suspected or confirmed cases have been reported in the past month, 1.4) Work in quarantine facilities.

2) COVID-19 infection is suspected by treating physician.

Clinics at the camps have developed screening questions at triage to ask all persons presenting for care about possible exposures, as outlined above. Medics or other NGO staff will evaluate if a patient meets the symptom and exposure criteria to meet the scenario 2.1 case definition. Additionally, persons who are under quarantine because they are known to meet one of the above exposure criteria and who have symptoms would also be considered to meet scenario 2.1 case definition.

b. Scenario 2.2: Patient with pneumonia suggestive of COVID-19 by clinician where one of the following criteria is met:

1) Severe conditions requiring intubation or death

2) Unknown causes or causes cannot be determined within 48 hrs.

3) COVID-19 infection is suspected by treating physician.

The Scenario 2.2 case definition approximately corresponds to the HIS case definition of "1. Severe Atypical Pneumonia": Acute severe lower respiratory tract symptoms requiring hospital admission with at least one of the following manifestations: inability to drink, frequent vomiting, convulsion, lethargy or unconsciousness, fever ≥38°C is not decreased after 3 days antibiotic treatment, requires referral to hospital outside of the temporary shelter, requires endotracheal intubation, death; however WITHOUT requiring the following epidemiologic exposures: Poultry OR other severe pneumonia case OR travel to country with known cases of Severe Acute Respiratory Syndrome (SARS) or pandemic influenza.

Additionally, Scenario 2.2 case definition can also be met by HIS case definition "6. Severe Case/Death of Unknown Etiology from any suspected cause of infectious diseases"

When PUIs that fit Scenario 2.1 or 2.2 are identified, NGOs will immediately isolate the patient and contact the district health officer and/or medical facility, and complete MOPH's Novelcorona 2 Form for referral to the nearest district medical facility. Alternately, specimen collection may be performed at the camp if referral is not available. More details of referral will be covered in section 3 of this document.

c. Scenario 3: Healthcare workers in hospitals, clinics, health promotion hospitals, laboratories, drug stores or disease investigation teams or personnel in quarantine facilities with any of the respiratory symptoms: cough, nasal mucus, sore throat, loss sense of smell, loss sense of taste, diarrhea, eye redness, rash, rapid breathing, shortness of breath, or dyspnea and/or with fever or temp ≥37.5°C.

Healthcare workers (including camp-based assistants and medic staff) will be similarly instructed to self-observe or be formally observed by NGOs each day for the above illness symptoms before they report to work if COVID-19 has been confirmed within the camp.

d. Scenario 4: Clusters of patients with acute respiratory infections ≥5 cases in the same place and same week with epidemiologic link.

The Scenario 4 case definition approximately corresponds to the HIS Case definition of "7. Influenza Like Illness": Fever >38 °C with at least two of the following signs/symptoms: sore throat, cough, runny nose, myalgia (muscle pain). Currently, thresholds are set for ILI outbreak declaration, that is based on historical average numbers of cases reported per camp per week. Currently, ILI in health care workers is not specifically tracked.

For the purposes of using ILI surveillance for COVID-19 detection, the following standard threshold has been set for reporting and investigation in the shelters by MOPH: If above weekly ILI threshold, 10% of ILI cases will be randomly

selected for COVID-19 test. As an example, if the ILI threshold in a given week is 45 cases and there are 50 reported ILI cases, 5 cases will be randomly selected for testing. Nasopharyngeal swabs will be collected and tested for COVID-19.

Probable: PUI with positive result from SARS-CoV-2 antigen test kit (ATK), where RT-PCR has not been performed.

Confirmed: PUI with positive result from SARS-CoV-2 RT-PCR from laboratory that has been certified by Department of Medical Sciences or from sequencing or culture. (List of certified laboratories for COVID-19 test as of August 4th, 2021: https://service.dmsc.moph.go.th/labscovid19/backend/uploads/20210804065806.jpg, https://service.dmsc.moph.go.th/labscovid19/).

Asymptomatic infection: Person with positive result from SARS-CoV-2 RT-PCR from laboratory that has been certified by Department of Medical Sciences or from sequencing or culture but without and signs or symptoms.

ii. **Enhanced Surveillance**

Patients meeting the CCSDPT Health Information System case definition for Influenza-Like Illness (ILI) and a proportion of patients meeting case definition for Acute Respiratory Infection (ARI) will be offered COVID-19 testing. These patients will be identified as part of routine ARI/ILI surveillance in the camps. For patients meeting the ILI or ARI case definition, they will be encouraged to self-isolate at home while having symptoms and there will not be any contact tracing.

Depending on the overall number of patients with ILI/ARI diagnosed per week, a random proportion of at least 10% of patients with ARI may be selected, based on the total number of patients with ARI presenting to the clinic in a given day (or period of 2 days if fewer than 10 persons with ARI are seen at the clinic per day). The rationale for selecting 10% of ARI patients is because generally the clinics see a large number of patients meeting these criteria, and so it would be impractical to test all patients. Logbook selection process for 10% of ARI patients: Every 10th patient diagnosed with ARI as written in the clinic logbook will be selected for COVID-19 testing. However, during the

surge of cases in the province where the camps are located, 100% of ILI/ARI diagnosed patients should be tested for COVID-19 either by antigen test kits (ATK) or RT-PCR. According to the 2019 HIS Case Definitions (UNHCR), ILI and ARI are defined as follows. Please note that ARI includes both Upper and Lower Respiratory Tract Infections (URTI and LRTI, respectively):

1. **Influenza-Like Illness** (ILI): Adult or child with temperature ≥38°C or subjective fever; and cough or sore throat or runny nose; and does not meet criteria for Lower Respiratory Tract Infection (LRTI) or person under investigation

2. **Upper Respiratory Tract Infection** (URTI): Adult or child with cough/cold (non-pneumonia) with running nose, cough, and low-grade fever.

3. **Lower Respiratory Tract Infection** (LRTI) in ages 5 years or older with temperature ≥38°C or subjective fever; and cough or sore throat; and breathing rate >20 breaths per minute

iii. **Surveillance during resettlement medical exams**

Displaced persons who are scheduled to travel from the camps to the International Organization for Migration (IOM) clinic for resettlement medical examinations and other similar activities will continue to be monitored for illness as follows. Prior to leaving the camp, IOM staff will perform exit screening by taking the person's temperature and verifying that they do not have any symptoms of COVID-19 through oral report. While the person is away from the camp, each day the temperature and symptoms will be similarly checked.

a. If a person outside of the camp for resettlement develops an illness consistent with the case definitions for PUI, IOM will communicate to the Health NGO at the camp about the illness and to the Mae Sot DHO using MOPH's Novelcorona 2 Form. Mae Sot DHO will likely accept the PUI for referral and testing. When referral is not available, IOM will isolate the PUI patient at a dedicated isolation facility, collect a naso-pharyngeal (NP) specimen for SARS-CoV-2 RT-PCR testing, and monitor the patient daily.

(i) If the test result is positive, IOM will inform Health NGO and DHO immediately once the result is known, and refer to the patient to district health facility as per MOPH guideline.

(ii) If the test is negative, IOM will inform Health NGO and DHO within three hours, and the relevant parties will negotiate a plan for patient care appropriate for the situation on a case-by-case basis.

b. If the ill person meets the case definition for ILI or ARI, IOM will notify the Health NGO at the camp about the illness, isolate the patient, and collect an NP specimen for SARS-CoV-2 RT-PCR testing as part of enhanced surveillance. Once a negative test result has been obtained and patient symptoms have improved, IOM will coordinate with Health NGO for the patient's return to the camp and for further follow-up as needed.

iv. **Community Based Surveillance:**

NGOs administer additional surveillance outside of the healthcare facilities by volunteer community members, and healthcare staff, who will observe for symptoms of COVID-19, advise on prevention and sanitation measures, and refer for care.

v. **Case Reporting to Surveillance**:

a. PUI Reporting: PUIs who meet the MOPH Scenario 2.1, 2.2, 3, or 4 Case Definitions will be reported to the District Health Office, who will then contact the Provincial Health Office, and PHO will notify the regional Office of Disease Prevention and Control (ODPC) according to guidelines on the DDC Web site (Appendix Figure). DHO will generate a 'patient ID number' for each confirmed case and then upload data online for specimen collection and COVID-19 lab test (see diagram below). In the case where laboratory testing is unavailable, the DHO will still be made aware that laboratory testing is pending but the specimen will instead be sent to a non-MOPH laboratory.

If a patient is outside of the camp at the time that the PUI case definition is met (i.e., diagnosed by IOM during resettlement examination), IOM will notify the

district health office and the Health NGO. The Health NGO will then notify the camp's district health office.

In the weekly OAF reporting, cases that meet with criteria in PUI case definition will be reported under Severe Atypical Pneumonia category of "Alert based on absolute value" and mention PUI COVID-19 in parenthesis next to the Severe Atypical Pneumonia. Case line listing is mandatory to fill out in the relevant section of the OAF. The special OAF is sent to relevant government health officials and stakeholders not later than 3 hours from the time of detection of the case.

b. Confirmed case reporting: Coordination with the DHO and PHO is needed for all reports of confirmed cases (including asymptomatic infection), to ensure that the case is only reported once. Any laboratory confirmed cases will be reported in the OAF under the severe atypical pneumonia disease category and mentioned clearly as Confirmed COVID-19 in parenthesis as mentioned above. The HIS surveillance focal person in the camp would complete the form and share with the HIS network as per described reporting procedures, and the case would also be included in the Weekly report. In the case where laboratory testing by the MOPH laboratory is unavailable for a PUI, the DHO will be made aware that laboratory testing is pending and the specimen will instead be sent to a non-MOPH laboratory. If the laboratory testing is performed in a non-MOPH laboratory, the NGO will immediately notify the laboratory result once it is reported.

c. If a patient is laboratory confirmed while outside the camp for resettlement examination with IOM, IOM will immediately notify the district health office and the Health NGO of the positive test results. The Health NGO will then immediately notify the camp's district health office.

3. Care Provision

i. **Temporary isolation at NGO facilities**—PUIs will be immediately isolated when they are identified. Patients presenting with ILI symptoms will also be separated from other patients to the extent possible. NGOs have limited temporary isolation capacity, for initial patient holding while referral is pending. To the greatest extent possible, NGO facilities

in-camp should not be considered the primary location to provide care for COVID-19 patients, due to limitations of infection control and potential for severe complications of the patients.

ii. **Referral to MOPH facilities**—As per MOPH guidelines and depending on local guidance, PUIs meeting the criteria for severe pneumonia or lower respiratory tract infection (MOPH Scenario 3) will be referred and isolated while test results are pending. Patients with milder symptoms may also be referred for isolation and testing, especially if they have a travel history to a high-incidence area or are a high-risk contact of a confirmed case (MOPH Scenario 2.1). The referral management will be discussed on case by case basis with the responsible staff at the district level. NGOs will request referral at the time of reporting to the District Health Office/receiving hospital and transfer patient information as per routine procedures for referral. The district health facility is the primary location for referral. Referrals to other districts are only done in special circumstances.

Confirmed cases from shelters may be referred for treatment at local government hospital. However, waiting time may vary which depends on the capacity and availability of the hospital at that time. Temporary shelters should prepare suitable isolation rooms for patients and close contacts when referral cannot be arranged. In the case that district isolation capacity is overwhelmed, NGOs provide in the camp at the isolated area by working with local authority to set up temporary isolation area in addition to existing facility.

4. Laboratory Testing

i. **Testing at MOPH network laboratories.** Testing of PUIs who have been referred to a district hospital or patients with ILI/ARI at the camp clinic will be routed through the Province or District hospital laboratory network. Specimens will be collected, stored, and transported as per hospital procedures. Positive results will be reported by the laboratory through the MOPH network to the District hospital, and the result will be communicated to the NGO by the District Health Official.

ii. As per MOPH Department of Medical Sciences requirements, aggregate counts of specimens tested in the following categories must be provided by the laboratories: PUIs,

Contacts, SQ/ASQ, and Sentinel Surveillance. Testing in the camps will be reported to the laboratories as follows:

a. PUIs—patients meeting PUI case definition

b. Contacts—patients meeting close contact definition

c. SQ/ASQ—people in travel quarantine who are tested

d. Sentinel Surveillance—people with ARI or ILI being tested as part of enhanced surveillance

iii. Testing at non-MOPH network laboratories. Testing of PUIs or patients with ILI who have not been referred to a district hospital (either because they do not meet criteria for referral or referral is not available) may be tested by a laboratory outside of the MOPH network. Specimen collection in the camp, storage, and transportation will follow SMRU and/or TUC specifications. As per request, a Novelcorona 2 form and an SMRU Microbiology or CDC COVID-19 specimen shipment form will be completed for each patient and submitted to the laboratory.

iv. **Positive** RT-PCR laboratory results by a non-MOPH laboratory will be reported first to the Provincial Health Office by the laboratory, and then the laboratory will directly contact the designated NGO point of contact. The NGO will and immediately send the Novelcorona 2 Form to PHO, DHO and stakeholders. If negative, IRC will report in the weekly outbreak alert form (OAF) and distribute to stakeholders as usual.

v. **Inconclusive** RT-PCR results occur on occasion when one RNA target is detected and another RNA target is not detected. This can happen when a patient has very small quantities of viral RNA or can result from laboratory error. The recommendation is for an additional specimen to be collected and for RT-PCR to be repeated. While awaiting a repeat test result, the patient should be treated as highly likely of being a true positive result—isolate and consider starting contact tracing. The repeat RT-PCR test result should be treated as the final result, as long as it is not inconclusive. Therefore, if the repeat RT-PCR test is negative, the patient will be not considered a case any longer and will not be reported as a case. If the repeat RT-PCR test is positive, the patient will be

considered a confirmed case. If the repeat RT-PCR test is inconclusive, another specimen should be collected and tested until a final conclusive result can be obtained.

vi. **Antigen test considerations**: Antigen test kits (ATK) may be used in certain settings in the refugee camp setting. These tests however must be treated with caution because they are not as sensitive (there can be false-negative results) and are not as specific (there can be false-positive results. Generally, false-negative results are most commonly encountered.

Antigen tests can be used effectively in the following scenarios:

- Testing a highly suspect PUI or high risk contact, where a positive result can lead to immediate isolation while RT-PCR results are pending (ie, reducing exposure risk of other people)

- Screening purposes when an outbreak has already been detected in the general camp community, such as screening before medical processing, active case finding, or contact tracing

RT-PCR testing is preferred for testing in the following situations:

- Sentinel surveillance when an outbreak has not been detected in the general camp community, to reduce the risk of false positives from ATK

- Contact tracing when the contact is potentially infectious and in contact with others

Following up antigen testing with RT-PCR is a good strategy to also reduce inaccurate test results, and PCR testing should be sought in the following situations:

- A patient with symptoms and known exposure tests negative by antigen test

- A patient with no symptoms and no known exposure tests positive by antigen test

Thai MOPH guidelines advise that all patients with antigen-positive test results should be confirmed by RT-PCR, and their case status is considered "probable" until RT-PCR result. (List of ATK approved by Thai FDA: https://www.fda.moph.go.th/sites/Medical/SitePages/test_kit_covid19.aspx)

vii. For high-risk contacts of cases, MOPH recommendations are to have a specimen collected for RT-PCR testing at day 5 after last contact with a confirmed case to facilitate early

detection, if they have not developed symptoms before this time. Symptoms will be assessed by the NGO contact tracing teams and specimens collected to follow this scheme.

viii. Healthcare workers who have been providing care for COVID-19 cases without appropriate PPE will be classified as high-risk contact. They will have to do self-quarantine for 14 days and nasopharyngeal swab should be collected for COVID-19 testing at day 5 after last contact regardless of their symptoms.

5. Case Investigation

As soon as a PUI is identified, NGO investigation teams will lead the process of interviewing the PUI and identifying contacts, with assistance provided by District Health Office.

Investigation teams will interview PUI (using Novelcorona 2 form) and relatives for:

• Travel history from countries [or parts of Thailand] with ongoing outbreaks or contact with persons who had travel history outside of the camp.

• For PUI without travel history, ask about recent healthcare visits or exposure to known COVID-19 cases within 14 days before onset.

• Other exposures such as contact with other patients (type, duration and frequency of activities) within 14 days before onset.

For each close contact identified, a Close Contact Identification form will be completed and all identified contacts will be listed on a contact listing form. This list will be used as the basis for contact tracing.

Close contact definition: an individual who had been with confirmed cases either during 14 days before symptom onset, or from the date of onset until the patient was isolated.

High risk close contacts are persons who had any of the following exposures:

• Talked to patient within 1 m for more than 5 minutes or exposed to cough/sneeze from patient while not wearing PPE according to standard precautions

• Stay in closed area with patient without good ventilation such as air-conditioned bus, room or being close to patient within 1 m for more than 15 minutes without protective equipment.

• Family members, relatives or caregivers for symptomatic COVID-19 patients

• Live in the same house with COVID-19 patients

• Meet with symptomatic COVID-19 patient **and** history of exposure to respiratory excretion or cough/sneeze from patient.

• Healthcare workers without appropriate PPE

• Other patients (with other medical conditions) who are/were hospitalized during the same period as, in the same room as, in the same row as the COVID-19 case, and visitors of those patients who visited the patients when the COVID-19 case had yet to be moved to an isolation room.

• Laboratory staff who did not wear PPE according to standard precautions while handing and processing clinical specimens collected from COVID-19 case.

• Students or co-workers include close friends who were interacting or mingling with symptomatic COVID-19 case; AND who may have been exposed to respiratory secretions, cough, sneeze from COVID-19 case

• Individuals who live in the same community as COVID-19 case or in another community, AND who have been exposed to respiratory secretions, cough, sneeze of the case

• Passengers who were exposed to respiratory secretions, cough, or sneeze from the case

• Co-travelers in the same group as the case, passengers in the same tour group

• Passengers on board the same flight as the case (in the same row as the case, and in the immediate 2 front and back rows) where masks were not worn for >5 minutes.

• Passengers on the same bus. In case of larger vehicle, e.g., train or double-decker buses or ferries, limit to only passengers within the same compartment or section where masks were not worn for >5 minutes

• All drivers and attendants, except for flights, include only crew in the same section of the plane where the case was seated, where masks were not worn for >5 minutes

Low risk close contact

• Hospital staff, laboratory staff, whose job is related to COVID-19 case, or visitors of hospitalized patient, who were wearing PPE according to standard precautions.

• Students or co-workers who are in the same class/room/department as symptomatic COVID-19 case who do not meet the definition of high-risk close contact.

• Individuals who live in the same community as COVID-19 case and were found to be interacting with symptomatic case of COVID-19 within one-meter distance.

• All passengers in the same vehicle as the confirmed case who do not meet the criteria of high-risk contacts. Remark: In case of larger vehicle, e.g., train or double-decker buses or ferries, limit to only passengers within the same compartment or section.

6. Contact Tracing

Contact tracing and quarantine of high-risk contacts will begin *before laboratory confirmation of PUIs* because it is anticipated that there may be delays in receiving laboratory test results. When laboratory results show that the PUI does not have COVID-19, then the quarantine and contact tracing for these persons will discontinue. NGOs will oversee monitoring of high risk contacts and administer a 14 day quarantine. In the event that a contact develops symptoms and becomes a PUI, NGOs will immediately report the PUI, place into temporary isolation, and contact with the responsible staff at the district level for further management. NGOs will also advise social distancing and self-monitoring, reporting of symptoms, and ensure use of face masks for all low-risk contacts.

Management of high risk close contact

• Quarantine for 14 days from the day of last contact with patient at the specified area.

• Designated staff check for symptoms and body temperature every day

• If resources allow, specimen collection and test at 5 days after last exposure, and retest 7 days after first test.

Management of low risk close contact

• Resume normal life activity. Avoid crowded area. Perform self-monitoring for 14 days from the day of last contact with patient.

• If develop fever or respiratory symptom, notify health officer immediately for specimen collection. Monitor sign and symptom, body temperature as high-risk contact.

7. Quarantine

i. **Persons with a History of Travel** In accordance with MOPH quarantine requirements, all persons with travel history outside of Thailand in the last 14 days will be subject to quarantine and testing. Additionally, camp residents with travel history to a province within Thailand that has reported COVID-19 cases in the past 14 days will also be subject to quarantine and testing.

ii. Criteria should be clearly communicated to all camp residents, camp staff, and District and Province Health Officials. Guidance will be administered by the NGO and the quarantine will be overseen by camp committee or camp commander, with the option of the community/camp providing quarantine facilities if the contact's household is not suitable for home quarantine. Camp committees will also ensure that persons under home quarantine will have necessary supplies available to ensure compliance.

Given the constraints in the camp setting and the lag time to transport specimens and receive laboratory results, this schedule will be adjusted so that specimens will be collected from persons two times during quarantine—first specimen on day 0–6, and second specimen on day 7–10. All persons in quarantine will also be observed daily for symptoms of COVID-19 and will be tested if they meet PUI criteria as previously described.

Quarantine facilities in the camp setting are also constrained in terms of space, and so individual rooms and bathrooms may not always be available for use. As such, Thai MOPH guidance for "Organizational Quarantine" (published September 8 2020) includes the following standards: If individual housing is impossible, limit to no more than 10 persons per room, with 1.5–2 m between beds, plus wearing masks all the time (except while asleep), social distancing, frequent handwashing, and avoid talking to others or gathering in group. If confirmed case is found:

o refer confirmed case to paired hospital

o quarantine all close contacts for at least 14 days, from the day of last known contact or lab confirmation

o halt all activities among high risk contact, quarantine individually or deport to country of origin

iii. **Contacts with High Risk Exposure to PUIs where laboratory testing is pending and laboratory confirmed COVID-19 Cases**—Due to high likelihood of high risk contacts developing COVID-19 and difficulties with administering self-quarantine, ideally quarantine facilities will be provided by the NGOs or by Camp commanders or shelter resident leaders with technical guidance from Health NGOs with adequate social distancing and daily direct follow up to observe for symptoms. In some situations, home quarantine may be necessary for contacts of PUIs or confirmed cases due to unavailability of separate facilities. If home quarantine, health volunteer or designated staff should visit those high risk contact every day to monitor their temperature and symptoms.

iv. **Healthcare workers providing care to COVID-19 Cases**—NGOs may institute a rotation schedule for their healthcare worker staff who are providing care to COVID-19 cases, where they live separately from their families for a designated “shift” while they are actively providing care, and afterward follow a 14-day quarantine period before returning to their households to avoid exposure to non-healthcare worker family members.

8. Conclusion of the Outbreak

It is an indication that an outbreak may be ending when it has been longer than one incubation period (14 days) since the last case was confirmed. Surveillance and contact tracing activities will continue for an additional incubation period from this point until it has been 28 days from the last confirmed case. At this time, the outbreak will be considered closed.

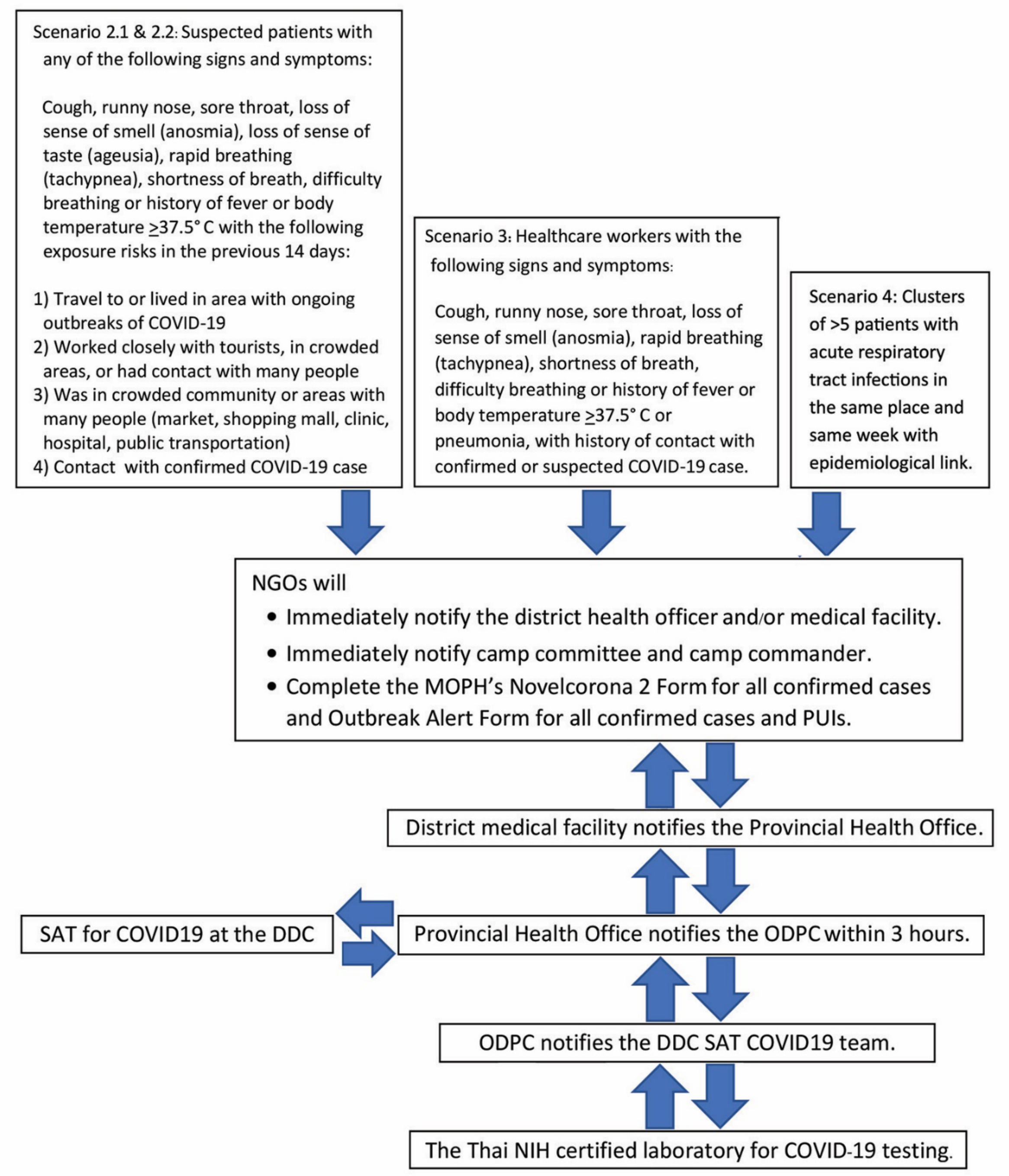

Appendix Figure. Reporting schemes for PUIs in temporary shelters for displaced persons, Thailand–Myanmar border, 2020–2021. If laboratory testing is unavailable, the DHO will still be made aware that laboratory testing is pending, but the specimen will instead be sent to a non-MOPH laboratory. If the laboratory for PUI is done in a non-MOPH laboratory, the NGO will immediately notify the laboratory result once it is reported. DDC, Department of Disease Control; MOPH, Ministry of Public Health; NGO, nongovernmental organization; ODPC, Office of Disease Prevention and Control; PUI, Person Under Investigation; SAT, Situation Awareness Team.
